# A Health App Platform Providing a Budget to Purchase Preselected Apps as an Innovative Way to Support Public Health: Qualitative Study With End Users and Other Stakeholders

**DOI:** 10.2196/49473

**Published:** 2023-09-29

**Authors:** Romy Fleur Willemsen, Eline Meijer, Liselot Nicoline van den Berg, Luuk van der Burg, Niels Henrik Chavannes, Jiska Joelle Aardoom

**Affiliations:** 1 Department of Public Health and Primary Care Leiden University Medical Center Leiden Netherlands; 2 National eHealth Living Lab Leiden University Medical Center Leiden Netherlands

**Keywords:** eHealth, health apps, health app platform, prevention, public health, health platform, health promotion, digital health, app evaluation, Framework Method, focus group, focus groups, evaluate, evaluation, platform, acceptability, feasibility, mHealth, mobile health, app, apps, application, applications, mobile phone

## Abstract

**Background:**

eHealth has the potential to improve health outcomes. However, this potential is largely untapped. Individuals face an overload of apps and have difficulties choosing suitable apps for themselves. In the FitKnip experiment, individuals were given access to a health app platform, where they could purchase reliable preselected health apps with a personal budget of €100 (US $107.35). By conducting a prospective study, we aimed to scientifically evaluate the FitKnip experiment as an innovative way to improve population health.

**Objective:**

The aim of the experiment was to scientifically evaluate the FitKnip experiment as an innovative way to improve population health. More specifically, we conducted an in-depth qualitative evaluation of the concept and acceptability of FitKnip, its perceived impact on health empowerment, as well as the roles of stakeholders for the future implementation of a health app platform through focus group interviews.

**Methods:**

This study followed a phenomenological research design and included 7 focus group interviews with end users and 1 with stakeholders, held between July and December 2020. End users were recruited through various institutions in the Netherlands, for example, insurance companies and local governments. All focus groups were semistructured using interview guides and were held via videoconferencing due to the COVID-19 pandemic measures. Each participant received access to a health app platform where they were enabled to purchase reliable, preselected health apps with a budget of €100 (US $107.35). The budget was valid for the entire research period. The health app platform offered 38 apps. A third party, a health care coalition, selected the apps to be included in FitKnip. The analyses were conducted according to the principles of the Framework Method.

**Results:**

A priori formulated themes were concept, acceptability, health empowerment, and outcomes, and the roles of stakeholders for the future implementation of a health app platform. Both end users (n=31) and stakeholders (n=5) were enthusiastic about the concept of a health app platform. End users indicated missing apps regarding physical health and lifestyle and needing more guidance toward suitable apps. End users saw health empowerment as a precondition to using a health app platform and achieving health outcomes depending on the purchased mobile apps. End users and stakeholders identified potential providers and financing parties of FitKnip. Stakeholders recommended the establishment of a reputable national or international quality guidelines or certification for health and wellbeing apps, that can demonstrate the quality and reliability of mobile health applications.

**Conclusions:**

This study showed the need for a personalized and flexible platform. Next to this, a deeper understanding of the roles of stakeholders in such initiatives is needed especially on financing and reimbursement of health promotion and digital health services. A personalized, flexible health app platform is a promising initiative to support individuals in their health.

## Introduction

A shift from the treatment of diseases to health promotion and prevention is necessary to overcome the challenges the health care system currently faces, namely increasing health care costs [1,2] and limited human resources [3,4]. eHealth defined as “the field of knowledge and practice associated with the development and use of digital technologies to improve health” has the potential to improve health outcomes [5]. The amount of health apps is on the rise, with over 90,000 health apps joining the market in 2020, leading to a total of over 350,000 health apps available in app stores [6].

However, the potential of eHealth to support health promotion and prevention is largely untapped [6], as long as the uptake of health apps in practice stays behind. Barriers to the successful uptake of eHealth mentioned by individuals and eHealth experts are a lack of: regulations around privacy and security, scientific evidence on the effectiveness and efficacy of these apps, evaluation of quality, and clarity with regards to financing [7-11]. Moreover, individuals face an overload of apps and have difficulties choosing suitable apps for themselves [12]. Specifically for paid apps, individuals are hesitant, since they do not know beforehand whether the application is worth buying [13]. This overload calls for guidance toward safe and effective health apps for individuals by regulation, application certification processes, [7,11] or, as suggested by van Velsen et al [12], “gateway apps,” which provide reliable health information or refer to other reliable health apps. The abovementioned barriers need to be overcome to stimulate the uptake of evidence-based, secure, and high-quality apps and therefore fulfill the potential of eHealth for health promotion across the population.

These barriers also exist in the Netherlands [12,14] and the uptake of health apps differs per group, more specifically, there is a higher uptake among younger, female, and highly educated individuals [14]. The Dutch government has ambitious goals for advancing the digitalization of care and stimulating the use of eHealth and health apps, for example, by financing and promoting eHealth and health apps [15]. Therefore, a national experiment was set up to make evidence-based, secure, and high-quality health apps accessible to individuals. In this experiment, individuals from the general population were given access to a health app platform “FitKnip” (FitKnip is a combination of Fit = being fit and the Dutch word “Knip” = wallet), where they were able to purchase reliable preselected health apps with a personal health budget of €100 (US $107.35). FitKnip was initiated by The Dutch Ministry of Health, Welfare and Sport. The selected apps focused on positive health, incorporating physical and mental health as well as social well-being, and not only the absence of disease. There are six central themes based on the dimensions of “positive health”: (1) bodily functions, (2) mental functions and perception, (3) spiritual or existential dimension, (4) quality of life, (5) social and societal participation, and (6) daily functioning [16]. The experiment was aimed to stimulate the uptake and usage of eHealth and to empower individuals to work on their health and vitality, ultimately supporting a more healthy society. By conducting a prospective study, we aimed to scientifically evaluate the FitKnip experiment as an innovative way to improve population health. More specifically, we conducted an in-depth qualitative evaluation of the concept (ie, what did the participants think about the idea of preselected applications and the budget) and acceptability (ie, did the participants find it easy or hard to use the platform and why?) of FitKnip, its perceived impact on health empowerment (ie, what helps participants in using the platform and how does this influence their health empowerment), as well as the roles of stakeholders for the future implementation of a health app platform (who should implement and finance a health app platform according to the end users and other relevant stakeholders?) through focus group interviews.

## Methods

### Study Design

**Figure 1:**
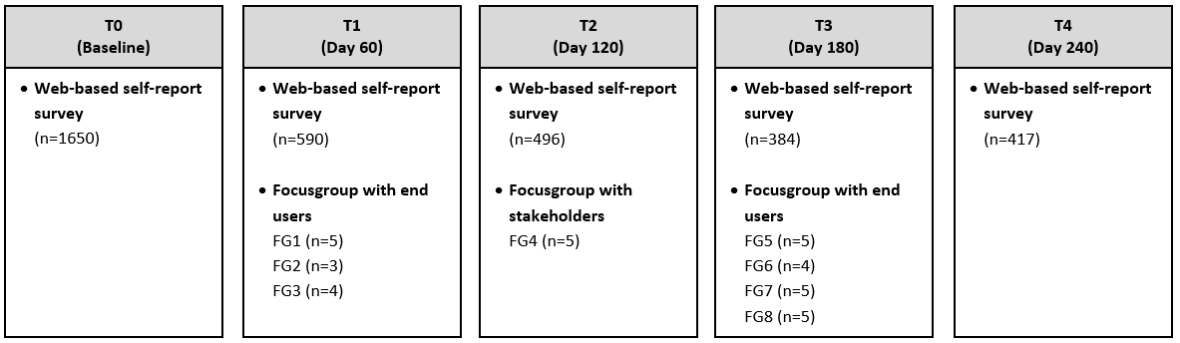
Overview of the study design of the mixed-methods study investigating FitKnip.

This study had a prospective interventional design and was part of a mixed methods study, referred to as the “FitKnip study.” The study consisted of 5 measurements over an 8-month period (see Figure 1), assessing feasibility, acceptability, health empowerment, and preliminary health outcomes. Outcomes were assessed quantitatively (ie, surveys and digital usage data of the FitKnip platform) and qualitatively (ie, focus group interviews). The qualitative part of the study, which we report on in this paper, followed a phenomenological research design since the aim of the study was to describe the experiences of the target group with respect to the health app platform. The study included 7 focus group interviews with end users at T1 (±60 days after baseline) and T3 (±180 days after baseline), and 1 focus group interview with other relevant stakeholders in the field of eHealth at T2 (±120 days after baseline). The focus groups with end users were held on 2-time points to be able to evaluate potential differences in experiences with longer exposure to the platform. The focus group with other stakeholders was held once, since the stakeholders were only familiar with the general concept of the platform, but did not have actual access to the platform. This study is reported according to the COREQ (Consolidated criteria for reporting qualitative research) guidelines where applicable [17] (see Multimedia Appendix 1). The sample size was not predetermined: focus groups with end users were held until data saturation was reached. After the last focus groups, the research team discussed if new or relevant information emerged and if additional focus groups were needed.

### Ethical Considerations

The FitKnip study was declared to not fall within the scope of the Dutch Medical Research Involving Human Subjects Act by the medical ethics committee of Leiden, Den Haag, Delft (N19.0878).

### Study Population

A total of 2562 participants (potential end users) were recruited for the FitKnip study through various institutions in the Netherlands, including health insurance companies, a health care coalition, an academic hospital, the Ministry of Health, Welfare and Sport, one of the largest employers’ organizations, local governments, municipality teams, a knowledge and quality institute for oncological and palliative care, as well as an digital platform for patients with cancer and their caregivers. Both on- and offline recruitment methods were used. A total of 1650 end users filled out the T1 questionnaire. End users were invited for focus groups based on their self-reported baseline sociodemographic and clinical characteristics (ie, age, sex, education- and work status, and medical or mental diagnosis [Do you have a medical or mental diagnosis? Yes or No]), in order to strive for a heterogeneous study sample (purposive sampling). Relevant stakeholders in the field of eHealth were recruited via purposive sampling (on job title) within the network of the National eHealth Living Lab. We aimed to include general practitioners (GPs), health care insurers, and policymakers.

Approximately 800 out of 1650 end users (48%) indicated they were interested in participating in a focus group at T1, and 56 out of 384 (14.6%) at T3. Between July and December 2020, 8 focus groups were conducted with end users and 1 with stakeholders, comprising a total of 31 participants (see Figure 1). More participants were scheduled for a focus group interview; however, 4 participants had to cancel on short notice, and 1 participant did not show up.

### Inclusion Criteria

The inclusion criteria for participation in the FitKnip study, including the focus groups, were (1) being 18 years old or older; (2) being able to understand, read, and speak the Dutch language; and (3) having access to the internet. An additional inclusion criteria for stakeholders is that they need to be currently employed as a GP, an employee of an insurance company, a policymaker on a national or municipality level, or as a community worker.

### Intervention: FitKnip

Each participant received access to a health app platform where they were enabled to purchase reliable, preselected health apps (mobile apps and progressive web apps) with a budget of €100 (US $107.35). Progressive web apps are web apps that offer mobile app–like experiences, while giving the user a faster and more reliable version of the app, for example, when having a poor internet connection. The budget was valid for the entire research period. The health app platform offered 38 apps.

The apps featured on the FitKnip platform were selected by a healthcare coalition acting as a third party. The selection criteria were: the app must be ready for use; can be used independently without involving a health care professional; should have no in-app purchases; the provider was willing to enable the process of anonymous purchases; was not allowed to gather, publish, or sell user data or use the data in any other way; and should adhere to national and international law applied to the FitKnip study, such as the General Data Protection Regulation.

### Procedure Focus Groups and Data Collection

The focus groups with end users were conducted in July 2020 (T1) and December 2020 (T3). The focus group with stakeholders took place in November 2020 (T2). The end users signed a digital informed consent before the start of the study and stakeholders signed an informed consent prior to the start of the focus group interviews. End users received reimbursement in gift vouchers of €25 (US $26.84) and stakeholders €75 (US $80.51).

All focus groups were semistructured using interview guides (see Multimedia Appendix 2). The focus group interviews were conducted via videoconferencing by author RFW, who was trained to do interviews and had experience in qualitative research. Since the focus group interviews took place during the COVID-19 pandemic, they were held via videoconferencing with several adjustments compared to face-to-face. More specifically, the focus group interviews were shorter in duration (90 minutes instead of 120 minutes or longer), contained a break and included less participants (4-5 instead of 9-12 participants) [18-21]. The focus groups were conducted via the videoconferencing platform Jitsi. Our experiences with these focus groups via videoconferencing, and our choices made throughout the preparation and execution phase, are described elsewhere [21].

### Data Analyses

All focus group interviews were audio recorded and transcribed verbatim for subsequent analyses. Names and other personal identifiers were replaced with participant numbers or removed to ensure participant confidentiality. The qualitative data analyses were conducted by authors LNvdB, LvdB, and RFW. Author RFW performed the final, integrative analytical steps of the analysis. The analyses were conducted according to the principles of the Framework Method [22,23], a systematic and flexible approach commonly used to analyze semistructured interview data. The method combines inductive and deductive techniques, which fit the aim of the present research to explore specific issues regarding patient experiences while leaving space to discover additional or unexpected patient experiences that have not been a priori formulated. The interview data were coded by LNvdB, LvdB, and RFW using the software Atlas.ti (version 9; ATLAS.ti Scientific Software Development GmbH). A coding tree, including themes and subthemes, was developed a priori based on the interview guide and was updated during the coding process whenever new themes, subthemes, or codes arose. We ensured the reliability of the analysis by having 5 focus group interviews (ie, 2 at T1, 2 at T3, and 1 with stakeholders) coded independently by authors LvdB (T1), LNvdB (T3 and the stakeholders focus group), and RFW (all). Following this, coded transcripts were discussed, and discrepancies were solved, which resulted in a final coding tree. Relevant quotes were brought together per code using the network function of Atlas.ti, categorized in various opinions. As a next step, it was explored whether the sociodemographic characteristics could potentially explain differences in opinions between end users or between stakeholders, for example, if younger end users have different opinions about the platform compared to older participants. Illustrative quotes presented in the Results section were shortened for length and clarity.

### Reflexivity Statement

The research team included researchers with varying levels of experience and diverse backgrounds (ie, public health and epidemiology, psychology, and medicine). All researchers had proficient digital skills, were healthy, and were highly educated. During the research, all researchers worked at the National eHealth Living Lab in the Netherlands and had expertise with, and had a keen interest in, eHealth.

## Results

### Sociodemographic Characteristics

End users participating in the focus group interviews (n=31) were on average 50 (SD 13; range 23-70) years old, mostly female (n=20, 64.5%) and highly educated (n=23, 74.2%), and 19 individuals (61.3%) had a medical diagnosis (Table 1). Compared with the total study population, end users in the focus group more often had a medical diagnosis; 12 (38.7%) versus 19 (61.3%), respectively. In the focus group for stakeholders, a total of 5 participants were included; a GP, an employee of an insurance company, policymakers on national and municipality levels, and a community worker.

**Table 1:** Sociodemographic and clinical characteristics of end users participating in the focus groups.

Characteristics		Values
Age (years), mean (SD)		50.3 (13)
**Gender, n (%)**		
	Male	11 (35.5)
	Female	20 (64.5)
**Education, n (%)**		
	Low	1 (3.2)
	Middle	7 (22.6)
	High	23 (74.2)
**Work status, n (%)**		
	Student	0 (0)
	Full-time employee	9 (29)
	Part-time employee	6 (19.4)
	Volunteer	2 (6.5)
	Retired	4 (12.9)
	Incapacitated	3 (9.7)
	Sickness benefit	4 (12.9)
	Other	3 (9.7)
**Diagnosis, n (%)**		
	Yes	19 (61.3)
	No	12 (38.7)

### Themes

The a priori-defined themes were concept, acceptability, health empowerment and outcomes, and future implementation (see Multimedia Appendix 3). The first theme, concept, is related to what end users and other stakeholders think about the idea of a health app platform. The second theme, acceptability, is related to end users’ experiences regarding the usability and acceptability of FitKnip. The third theme, health empowerment and outcomes, is related to end users’ perspectives on whether and how a health app platform has helped them or can potentially help them take control over their own health and achieve health goals. The last theme, future implementation, is related to end users’ and stakeholders’ perceived barriers and facilitators of future implementation of FitKnip, and the identification of possible parties to finance and provide health app platforms.

### Concept

#### Focus Groups With End Users

##### Positivity About the Concept and Budget

While not all end users provided their opinion on the concept or idea of the FitKnip platform, those who did express their views were quite positive about the health app platform. This positivity stemmed from the preselection of apps, the flexibility for end users to choose options that aligned with their needs, and the effective removal of financial barriers to acquiring health apps.

I find this an incredible initiative, since people are stimulated to use eHealth and money is no longer a barrier for using eHealth.

Female, 27—Focus group at T1

Most end users indicated that they considered the budget satisfactory since they could try out different apps. On the contrary, only 2 end users considered the budget too low, as they could not try out all the apps they were interested in.

I thought it was a super generous budget, and I tried a lot of different apps. I thought very carefully about what I wanted to do. The budget felt like a present.

Female, 47—Focus group at T3

##### End Users Highlight the Importance of Platform Transparency and Content Overview

A considerable part of end users would have liked more information and transparency on the selection criteria for apps, and a minority indicated missing information regarding which parties subsidized the budget provided within FitKnip.

A couple of end users indicated that FitKnip gave them an overview of the overwhelming amount of health apps; however, 2 participants expressed to still struggle to select an app fitting their needs. All end users who commented on whether FitKnip provided an overview of health apps had a diagnosis. A rationale behind this might be that people with health issues are more prone to look for health apps that help them manage their disease.

A couple of end users suggested that free apps and offline health programs may be added to FitKnip as this will provide more choices to end users to decide how they want to work on their health.

#### Focus Groups With Stakeholders

##### Positivity About the Concept and Budget

Stakeholders were enthusiastic about the concept and acknowledged the potential of FitKnip as a tool to support public health.

##### Better Fitting the Platform to the Needs of End Users

The community worker suggested FitKnip to be more useful when offline health programs would be offered as well, such as live sports classes, as he believed the effects of health apps alone would be limited. Especially since the apps provided within FitKnip were not suitable for the population in that neighborhood as apps were only available in Dutch and contained lengthy texts. In line with this, the GP added:

I have some trouble with the fact that non-Dutch speakers were excluded from this experiment. I notice a need for improvement of lifestyle in this population, indicated by both patients and healthcare professionals.

GP

In line with the feedback of end users, stakeholders considered the selection process of apps in FitKnip to be unclear. Furthermore, other questions arose, such as what were the exact selection criteria and why only paid apps were included on the platform. One of the stakeholders noticed that apps were selected mainly based on privacy regulations but considered this as a trade-off with the usability of apps.

### Acceptability

#### Focus Group With End Users

##### Improving Acceptability by Personalization and Better Guidance to Suitable Apps

In general, end users experienced the FitKnip platform as usable, neat, and orderly. They indicated that its usability could be increased by adding motivational reminders (eg, “You have not visited FitKnip in a while, how are you doing?”) and informative reminders (eg, “You have €30 (US $32.20) left to purchase apps”), preferably enabling personalization in terms of time and frequency. Next to this, end users indicated wanting to use 1 account for the platform and the apps, and that the platform links to the apps directly without having to sign on again.

Some end users were satisfied with the amount and type of apps. However, a similar amount of end users indicated missing applications regarding physical health and lifestyle, such as nutrition, sports, and exercise. These users were generally older (between 40 and 50 years) compared to end users who were satisfied with the apps provided by FitKnip (20-40 years). A reason for this might be that older users experience a greater inclination to work on their lifestyle compared to younger users. End users who had cancer or burnout indicated missing applications concerning mental capacity, fatigue, and consequences of cancer. Next to this, some end users suggested including apps in varying levels of expertise; beginner, intermediate, and advanced, meaning that there are, for example, beginner and advanced mindfulness apps.

End users mentioned several improvement opportunities for the platform to better guide them to potentially suitable applications for them. To start with, more information about the applications within FitKnip (eg, time investment or the app goal) could help to make a more informed choice. Another suggested way for improvement was to also offer information in the form of previews, reviews, or a short trial period. Moreover, end users indicated that next to the categorization in themes, they would like to be able to see categories of apps based on their corresponding target groups, such as age category, level of expertise, or stage of illness. Finally, some end users suggested that a personal recommendation for specific apps based on a couple of questions, functioning as a quick lifestyle scan, would help find a suitable app.

I think it would be helpful if I could fill out a couple of questions and that based on these results, recommendations for suitable health apps are being provided.

Female, 27—Focus group at T1

##### Privacy—Key Who Provides and Finances

Regarding privacy, a part of end users indicated seeing the platform as safe and trustworthy, especially due to the providers involved such as a medical university hospital or the local government. End users indicated experiencing less privacy in the applications offered within the platform. Additional information regarding the acceptability of FitKnip can be found in Multimedia Appendix 3.

### Health Empowerment and Health Outcomes

#### Focus Groups With End Users

##### Health Empowerment as a Precondition to Using a Health App Platform

A couple of end users perceived health empowerment as a precondition to deciding to use a health app platform and participate in the FitKnip experiment. Once having access to FitKnip, some end users indicated they experienced no improved health empowerment since the health app platform can provide support and insight, but not necessarily more control. Others indicated that FitKnip has the potential to support health empowerment but that the platform needs to be improved to fit their needs better. Especially with respect to the type of offered health apps, as some end users indicated having missed apps regarding physical health and lifestyle.

Considering health empowerment. I think we as a group are biased. We participate voluntarily. That already is an active step towards health empowerment. The apps are without obligation, so we choose what suits us. So in that sense, not more or less [health empowerment] than before. I guess the same.

Female 37—Focus group at T3

Nevertheless, a part of the end users indicated that FitKnip gave them more control over their health. Factors that end users named that stimulated this control were accessibility (ie, most of the apps could be used on a mobile phone instead of a computer), independence (ie, that they do not have to bother others with their problems), and being able to choose their own time and subject to work on their health. However, some end users indicated losing their usual structure as they stopped working due to retirement or illness. In that situation, it is difficult to work with FitKnip and potentially increase health empowerment; as 1 user explained:

When you talk about FitKnip and health empowerment, health empowerment is difficult. If you stop working [due to illness], you suddenly have a lot of time for these kinds of things. However, you can be easily distracted, and you don’t think about it for a while. So health empowerment is difficult in that sense.

Male 60—Focus group at T3 dec

##### Achieving Health Outcomes Depend on the Purchased Apps

End users were asked if and how the health app platform helped them achieve their health goals and what aspects facilitated or inhibited them from achieving them. A part of the end users indicated that FitKnip and the apps within FitKnip helped them create awareness and obtain information about health, and put this knowledge into practice.

I did “Stressles” [app], which is short and simple. I did not expect it, but I was helped tremendously as finally I was challenged to put the theory I already know into practice.

Female, 48 – FitKnip focus group T3

Other end users stated that they doubted whether FitKnip and the apps within FitKnip helped them achieve their health goals since some apps were of short duration, were too basic, or contained no new information. Barriers that were mentioned by end users in achieving their goals were a lack of discipline, motivation, time, and contact with others or stakeholders.

### Roles of Stakeholders for the Future Implementation of a Health App Platform

#### Advantages and Disadvantages of Possible Stakeholders to Provide and Finance a Health App Platform

End users and stakeholders perceived health insurers, primary or secondary care providers, patient associations, employers, or the municipality welfare workers as potential providers of FitKnip.

End users often mentioned the health insurer as a possible provider of FitKnip. They saw a role for the health insurer as an insurance company would have interest in an initiative such as a health app platform. However, other end users indicated being hesitant because of the commercial interest. The health insurer does not see a large role for health insurers in initiatives as a health app platform, as financing of prevention is not yet clear. The insurer indicated they could offer support in communication.

I would think of health insurers, as, assuming, everyone has health insurance [in the Netherlands], therefore everyone would have an account of FitKnip to stimulate health.

Male 53 focus group at T3

End users, as well as, stakeholders mentioned primary or secondary care as a provider. Both groups had doubts regarding costs; end users thought this would increase health care costs, and the GP emphasized another reimbursement system is needed once primary or secondary care is going to provide a health app platform.

End users and stakeholders saw the municipality as a possible provider of FitKnip. End users thought this was an accessible provider, as the municipality is close by and people can reach out easily. Stakeholders saw shared responsibility regarding prevention and saw the municipality as one of the potentially involved parties. More details on the results in terms of acceptability and future implementation can be found in Multimedia Appendix 4.

#### Trusted National Quality Mark

Next to identifying potential providers, stakeholders indicated that to facilitate implementation, they would like to see a trusted national quality mark to indicate the platform FitKnip and apps within FitKnip are proven effective.

## Discussion

### Principal Results

This qualitative study evaluated end users’ and other relevant stakeholder’s perspectives regarding a health app platform targeted at the general population to support public health. Both end users and other stakeholders were enthusiastic about the concept of this health app platform and the needs of end users and suggestions for improvement of the platform were identified. These suggestions showed the need for a personalized and flexible health platform, in terms of, for example, language options, app selection, and categorization of apps. Below, we compare the end users’ needs and improvement opportunities that can contribute to a personalized and flexible platform with current evidence and discuss practical implications.

### Comparison With Prior Work

To the best of our knowledge, this study is the first to evaluate the feasibility and acceptability of a health app platform (ie, a platform providing reliable preselected health apps with a personal health budget of €100 (US $107.35) with end user and other stakeholder experiences. However, participants in the study of Szinay et al [24] also found the concept of a curated health app portal appealing and indicated such a portal can alleviate privacy concerns and increase trust. Another health app platform has been studied, Intellicare, which was aimed at the treatment of anxiety and depression [25-27]. The users of this platform were shown to download more individual health apps compared to users who did not use the platform [25].

To start with, the current user population reported a need for a broader range of apps, especially ones targeting physical health and lifestyle, such as nutrition and exercise. This is in line with data of a large representative sample of the Dutch population showing that fitness and nutrition apps were most often installed on smartphones [14]. Some end users indicated that the current selection of apps did not seem to fit their goals and therefore did not use the platform. This is compatible with the unified theory of acceptance and use of technology, more specifically the performance expectancy, which refers to the degree to which an individual believes that using the technology will help them to achieve their goals. Performance expectancy is hypothesized to influence one’s behavioral intention to use certain technologies [28]. This underlines the need for a broader selection of apps, including apps about physical health, as this will increase the chance that an individual can find apps that will likely help them achieve their goals.

Second**,** the current results suggest that implementing reminders from the platform could help engage end users in using the platform and the individual apps, which is in line with the literature [13,29]. These reminders can motivate end users to use the platform and the purchased apps and can show information, such as the remaining budget. This addition especially helps if end users can set the frequency and timing of reminders themselves.

Third, our results indicated that language, literacy levels, and the selection of apps within the platform should match the abilities and needs of the end users. This is in line with a study by Bol et al [14], which showed that individuals’ gender and educational levels influence how and which health apps are used. Abilities and needs can vary over target groups, these suggest the need for a flexible platform that can be tailored to the specific needs of the target group.

Furthermore, a part of our end users indicated still having trouble selecting apps best suited to them and would like more guidance. Guidance for end users to select apps best suited for them can be offered in multiple ways, such as more information about apps before purchase, several ways of categorization of apps, and personalized advice. A health app platform helps in choosing an app in the current overload of apps [12]; however, some end users need additional guidance to find the most suitable apps for them next to the selection based on safety and privacy.

Finally**,** the platform itself was perceived as reliable due to the parties involved in FitKnip, such as the Leiden University Medical Center and the users’ employer. Individuals indicated having trust in these independent, noncommercial parties. In line with our study, Dennison et al [29] and Neher et al [30] established that the provider of a health app plays a role in trust end users have in the service. Apps developed by experts and provided by universities or public organizations are seen as more credible than apps from private parties or unknown, less reputable sources in general. This confirms the aspect of social influence, the degree to which an individual perceives that important others believe they should use the new system, to play a role in the usage and acceptability of eHealth [28].

Moreover, we explored perspectives on health empowerment in association with such a platform. Our results show that health empowerment is increased for some individuals and others see it as a precondition to use a health app platform. When empowering end users to work on their health and vitality, it is important to manage information flow to end users [31] and consider health literacy levels [32], to facilitate end users to make safe and informed decisions.

Focus group interviews were conducted at 2 measurement points, with the last one seeming to indicate increased enthusiasm among end users. We think end users who participated in the second measurement moment were more motivated to participate and might have been more enthusiastic about FitKnip from the start. Moreover, compared to the T1 focus groups, more end users with a diagnosis participated, which underlines the necessity of a platform for people with a medical diagnosis. Next to this, these users might be more willing to participate in the research, as during the focus group, these end users explained wanting to contribute to research in the field of their disease.

Facilitators and barriers to future implementation of the platform were identified regarding financing and providing a health app platform. Next, the need for a trusted national quality mark was recognized.

In this study, end users and other relevant stakeholders identified several possible funding parties for a platform like FitKnip. However, financing initiatives to stimulate health and well-being is complex since there is a gap in current reimbursement models regarding health promotion and digital health. Financial barriers to eHealth and mHealth implementation have already been largely documented [7,8,11,30]. Health care systems are shifting to value-based health care models, in which digital tools can have a dominant place, and reimbursement processes change in line with this; however, this development is going slow [33,34]. Health financing reforms need to accelerate; reimbursement and financing options for prevention, health promotion, and digital health need to be further explored.

Moreover, a need for a trusted national quality mark for both the health app platform and the apps within the platform was identified as a facilitator for future implementation. The need for eHealth services and app regulation and legislation is widely recognized [7,8,11,30,35]. A transparent selection process for apps within a health app platform will help end users in adopting and stakeholders in providing a health app platform. Guidelines and legislation regarding health apps and a standardized process for applying these guidelines will help future health app platforms.

### Limitations

To our knowledge, this is the first study to report on a public health experiment that provided citizens access to a health app platform where they were enabled to purchase reliable, preselected health apps with a budget of €100 (US $107.35). End users were allowed to try out the platform and corresponding apps for a period of 8 months, a relatively long period of time. Furthermore, end users were interviewed at 2 different time points within the project, thereby obtaining both their first impressions and their reactions after a more extended period of usage. Another strength is that perspectives from both end users and other relevant stakeholders were combined, enabling a broad perspective on interests and barriers for future implementation.

This study had several limitations. Although end users were recruited via different types of organizations (ie, health insurers, employers’ organizations, and patient associations), thereby striving for a heterogeneous population, our study population was still predominantly female and highly educated. Moreover, this study population had a mean age of approximately 50 years, and young adults (aged < 30 years) were vastly underrepresented in our study. Therefore, we have a limited perspective of young adults. Moreover, selection bias may have occurred as it is likely that individuals and stakeholders were already interested in, or using, eHealth and participated in our study. A limitation with respect to the focus group with stakeholders specifically is that we did not include all mentioned possible providers in the focus group interviews, that is, representatives from employer or patient associations. The understanding of future implementation could have been more complete if these stakeholders had been present in the focus group. Finally, due to the researchers’ characteristics, there is a possibility that they might not have fully grasped problems related to health and digital skills that participants were struggling with, which might have contributed to a more positive view of the health app platform.

### Conclusions

Both end users and other relevant stakeholders are enthusiastic about the concept of a health app platform to promote health and vitality. The current evaluation of the FitKnip platform provides insight into what end users and other stakeholders need, prefer, and envision for the future. To optimize the potential of such a platform to support health and health empowerment, the platform can be improved by tailoring it to the individuals’ specific needs and be customizable for individuals to tailor it to their needs. Next to this, a deeper understanding of the roles of stakeholders in implementing such an initiative is needed, especially in financing and reimbursement of health promotion and digital health services. A personalized, flexible health app platform is a promising initiative to support individuals in their health.

## Appendix

Multimedia Appendix 1 COREQ-checklist.

Multimedia Appendix 2 Interview guide.

Multimedia Appendix 3 Codebook.

Multimedia Appendix 4 Results acceptability and future implementation.

## Abbreviations

COREQ: consolidated criteria for reporting qualitative research
GP: general practitioner

## Data Availability

The data sets generated and analyzed during this study are not publicly available because participants have only granted consent for (1) the collection and use of their data for addressing the research questions only for this specific study and (2) their data only to be accessed by specific individuals (ie, the researchers) and entities if necessary to ensure the proper and reliable execution of the research. Hence, individuals have not consented to make their data publicly available.
