# Supplementary material for: A Health App Platform Providing a Budget to Purchase Preselected Apps as an Innovative Way to Support Public Health: Qualitative Study With End Users and Other Stakeholders
Source: JMIR Form Res. 2023 Sep 29;7:e49473. doi: 10.2196/49473 (PMC10576224; doi:10.2196/49473)
Supplement: Multimedia Appendix 1 [file formative_v7i1e49473_app1.docx]

**Domain 1: Research team and reflexivity**
**Personal Characteristics
1. Interviewer/facilitator Which author/s conducted the interview or focus group?**

The focus group interviews were conducted online by author RFW, who was trained to do interviews and had experience in qualitative research.

**2. Credentials What were the researcher’s credentials? E.g. PhD, MD**

MSc
**3. Occupation What was their occupation at the time of the study?**

PhD-candidate **4. Gender Was the researcher male or female?**

Female
**5. What experience or training did the researcher have? Relationship with participants**

RFW was trained to do interviews and had experience in qualitative research.

**6. Was a relationship established prior to study commencement?**

No relationship was established between the researcher and the participants prior to the study.

**7. What did the participants know about the researcher? e.g. personal goals, reasons for doing the research**

The participants new the occupation of the researcher, namely PhD-candidate.

**8. What characteristics were reported about the interviewer/facilitator? e.g. Bias, assumptions, reasons and interests in the research topic**

None

**Domain 2: study design
9. What methodological orientation was stated to underpin the study? e.g. grounded theory, discourse analysis, ethnography, phenomenology, content analysis**

The study had a phenomenological orientation.

**10. Sampling How were participants selected? e.g. purposive, convenience, consecutive, snowball**

Both for end-users and other stakeholders purposive sampling was used.

**11. Method of approach How were participants approached? e.g. face-to-face, telephone, mail, email**

The participants were approached via e-mail.
**12. How many participants were in the study?**

31 end-users and 5 stakeholders
**13. How many people refused to participate or dropped out? Reasons?**

Four participants had to cancel on short notice due to personal circumstances, and one participant did not show up.

**14. Setting of data collection Where was the data collected? e.g. home, clinic, workplace**

The interview were held online due to the COVID-19 crisis.

**15. Was anyone else present besides the participants and researchers?**

No, next to the researcher performing the interviews, 2 other researchers were present.

**16. What are the important characteristics of the sample? e.g. demographic data, date**

The participants were recruited from the general population.

**Table S1.** Socio-demographic and clinical characteristics of end-users participating in the focus groups.

| **Characteristics** | **Mean (SD) or n (%)** |
| --- | --- |
|  |  |
| **Age** | 50.3 (13.0) |
| **Gender**  Male  Female | 11 (35.5)  20 (64.5) |
| **Education**  Low  Middle  High | 1 (3.2)  7 (22.6)  23 (74.2) |
| **Work status**  1 Student  2 Fulltime employee  3 Parttime employee  4 Volunteer  5 Retired  6 Incapacitated  7 Sickness benefit  8 Other | 0 (0.0)  9 (29.0)  6 (19.4)  2 (6.5)  4 (12.9)  3 (9.7)  4 (12.9)  3 (9.7) |
| **Diagnosis**  Yes  No | 19(61.3)  12 (38.7) |

**Data collection**
**17. Interview guide Were questions, prompts, guides provided by the authors? Was it pilot tested?**

Yes, the questions, prompts and guides were provided by the authors in appendix A.

**18. Repeat interviews Were repeat interviews carried out? If yes, how many?**

Yes, 3 repeat interviews at T1 and 4 repeat interviews at T3. There was only 1 interview with stakeholders.

**19. Did the research use audio or visual recording to collect the data?**

The interviews were audio recorded.

**20. Were field notes made during and/or after the interview or focus group?**

No field notes were taken during or after the interview.

**21. Duration What was the duration of the interviews or focus group?**

Approximately 90 minutes.
**22. Was data saturation discussed?**

Yes, data saturation was discussed between RFW and JJA.
**23. Were transcripts returned to participants for comment and/or correction?**

No, but the audio recordings were transcribed verbatim.

**Domain 3: analysis and findings**
**Data analysis**
**24. How many data coders coded the data?**

3 in total. 2 the first round of interviews and 2 the second round of interviews and the stakeholder focus group.
**25. Did authors provide a description of the coding tree? 🡪 appendix?**

Yes, in Appendix 2.
**26. Were themes identified in advance or derived from the data?**

The themes were derived in advance.
**27. What software, if applicable, was used to manage the data?**

The data was managed in Atlas.ti version 9.

**28. Did participants provide feedback on the findings?**

No. participants did not provide feedback on the findings.

**Reporting**

**29. Were participant quotations presented to illustrate the themes / findings? Was each quotation identified? e.g. participant number**

Yes, quotations were presented to illustrate the themes and findings and each quotation is identified.

**30. Was there consistency between the data presented and the findings?**

Yes
**31. Were major themes clearly presented in the findings?**

Yes, the themes are clearly presented.

**32. Is there a description of diverse cases or discussion of minor themes?**

Yes
