# Supplementary material for: A Health App Platform Providing a Budget to Purchase Preselected Apps as an Innovative Way to Support Public Health: Qualitative Study With End Users and Other Stakeholders
Source: JMIR Form Res. 2023 Sep 29;7:e49473. doi: 10.2196/49473 (PMC10576224; doi:10.2196/49473)
Supplement: Multimedia Appendix 2 [file formative_v7i1e49473_app2.docx]

**Semi-structured Topics List Focus Groups (in Dutch) – Participants FitKnip**

*Aim: To identify possible improvements of FitKnip and the eHealth applications as part of FitKnip in terms of content, design, functionality, user-friendliness, privacy, reliability, complexity, type and number of offered eHealth applications. Moreover, to identify perceived barriers and facilitators for future implementation.*

1. **Introduction**
   1. Welcome
   2. Introduction topic
   3. Rules focus group
   4. Planning
   5. Informed consent
   6. Questionnaire
   7. Questions?
2. **Introduction round**
   1. (name, background)
3. **Opening topics**
   1. Explanation of FitKnip and what eHealth is.
   2. Did u already use eHealth prior to this study?
4. **FitKnip concept**
   1. In general, what do you think of FitKnip?
5. What do you think of the budget and why?
6. What do you think about providing reliable apps like this?
7. **FitKnip platform**
   1. What do you think of the applications provided within FitKnip?
8. What do you think of the themes in which the apps are categorized? Do you have additions?
9. What do you think of the selection of apps provided in FitKnip? Do you have additions?
10. Is the type of apps in line with what you expected? Why (not)?
11. What do you think of the amount of apps? Why do you think this is too less or too much?
    1. What do you think of the design of FitKnip?
    2. Considering design, what works well and what does not? Why?
    3. What do you find easy or hard in using FitKnip?
12. Why?
13. What does not work? Or what was not easy to find?
    1. What do you think is easy or hard in using the provided apps? Wat vindt u makkelijk of moeilijk aan het gebruik van de apps aangeboden in FitKnip?
14. Did you notice that for some apps you needed to register? What do you think is easy or difficult about that?
    1. What do you think is easy or difficult regarding the content of the provided apps?
15. What makes an app understandable or not?
16. **FitKnip health outcomes and privacy**
    1. Do you think the apps work? (for example an app about stress reduction acutually reduced your stress) Why (not)?
    2. Do you have the feeling your data is safe with FitKnip and the apps provided in FitKnip?
17. Do you worry about privacy? Why (not)?
18. How could this be improved?
19. What gives you less or more trust in privacy of a certain application?
20. **Skills and health empowerment**
    1. What helps you in using FitKnip? (for example. Skills to work with apps, time, motivation).
    2. Do you talk with others about FitKnip?
21. Your healthcare professionals?
22. Friends/family?
    1. What kind of influence does FitKnip have on your health empowerment?
       1. Why do you think this is?
23. **Implementation of FitKnip**
    1. What are barriers or facilitators fort he future implementation of FitKnip?
24. Why?
25. Do you think others’ also consider this a barrier/facilitator?
26. What could be a solution according to you?
27. **Closing**
    1. Of all the things we discussed today? What do you think is most important?
    2. Do you have additional comments/questions?
    3. Do you think differently about FitKnip then before this focus group?
    4. End of focus group
    5. Do you want to be informed on the results of the study?

**Appendix 1: Semi-structured Topics List Focus Groups (in Dutch) – Healthcare providers**

***Aim:*** *To identify perceived barriers and facilitators experienced by relevant stakeholders (i.e. healthcare professionals, health care insurance companies, employers’ organisations, local authorities, and legal parties) regarding a future implementation of FitKnip.*

1. Introductie, uitleg, informed consent
   1. Plaats: In de groep, niet aan het hoofd van de tafel
   2. Welkom. Introductie gespreksleider en notulist
   3. Inleiding onderwerp
   4. Regels focusgroep
   5. Planning
   6. Toestemmingsformulier
   7. Vragenlijst met basiskenmerken
   8. Reiskostenvergoeding
   9. Praktische vragen?
2. Voorstelrondje
   1. Naam, achtergrond
3. Openingsvragen
   1. Uitleg over FitKnip en wat eHealth over het algemeen inhoudt.
   2. Hoe bent u bezig met eHealth in uw werk?
4. **FitKnip strategie**
   1. Wat vindt u van het FitKnip concept?
5. Algemeen?
6. Wat betreft het budget?
7. Wat betreft de aangeboden apps?
8. Wat betreft de thema’s van de apps?
9. Wat betreft de doelgroep?
10. **FitKnip context**
    1. Denkt u dat de maatschappij behoefte heeft aan een strategie als deze? Waarom wel/niet?
11. Wat zijn meewerkende en tegenwerkende organisaties/thema’s hierbinnen?
    1. Hoe denkt u dat de deelnemers reageren/hebben gereageerd op FitKnip vanuit jouw perspectief?
12. Waarom denkt u dat?
13. Wat bepaalt volgens u of deelnemers positief of negatief reageren?
    1. Denkt u dat FitKnip aansluit op de behoeftes van uw beoogde doelgroep? (mensen in de wijken, patiënten die u behandeld, verzekerden etc.)
14. Op de behoeftes van welke individuen/groepen wel en welke niet?
    1. Wat zijn moeilijkheden waar deelnemers mogelijk tegen aan kunnen lopen bij het gebruiken van FitKnip?
15. Wat kan daar een oplossing voor zijn?
16. **FitKnip organisatie**
    1. Wat zou de rol van uw organisatie zijn wat betreft het implementeren van FitKnip?
    2. Welke aanpassingen zijn er in uw organisatie nodig voor het implementeren van FitKnip?
    3. Zijn er voldoende middelen aanwezig voor het uitvoeren en implementeren van FitKnip? (financiëel, privacy, technische ondersteuning, kennis, bereidheid)
    4. Voelt u zich gesteund vanuit de organisatie om deze veranderingen in gang te zetten?
17. **Uzelf/zorgverleners**
    1. Ziet u FitKnip als mogelijk middel om de gezondheidszorg te verbeteren?
    2. Hoe denkt u dat FitKnip terugkomt in uw werkzaamheden?
    3. Vind u FitKnip een goede aanvulling op uw werkzaamheden en waarom?
    4. Zou FitKnip uw bezigheden veranderen?
18. **Implementatie van FitKnip**
    1. Wat is er nodig voor een succesvolle implementatie van FitKnip?
    2. Welke partijen moeten er betrokken worden bij de implementatie van FitKnip? Waarom?
    3. Wat helpt u en wat werkt u tegen bij de implementatie van FitKnip?
    4. Heeft u voorbeelden van succesfactoren of tegenwerkende factoren van de implementatie van andere eHealth toepassingen, die ook op FitKnip van toepassing kunnen zijn?
19. **Afsluiting**
    1. Van al de dingen die we vandaag besproken hebben, wat vind u het belangrijkst?
    2. Samenvatting geven; Heb ik het zo goed samengevat? (overzicht op Whiteboard)
    3. Heeft u nog aanvullende opmerkingen/vragen? Of factoren die we nog niet besproken hebben?
    4. Bent u door de discussie anders gaan denken over FitKnip/eHealth toepassingen?
    5. Einde focusgroep
    6. Op de hoogte houden van uitkomsten onderzoek?
