# Supplementary material for: A Health App Platform Providing a Budget to Purchase Preselected Apps as an Innovative Way to Support Public Health: Qualitative Study With End Users and Other Stakeholders
Source: JMIR Form Res. 2023 Sep 29;7:e49473. doi: 10.2196/49473 (PMC10576224; doi:10.2196/49473)
Supplement: Multimedia Appendix 3 [file formative_v7i1e49473_app3.docx]

**Table S1.** Codebook focus groups with end-users.

| **Themes** | **Subthemes** | **Definition and coding rules** |
| --- | --- | --- |
| Concept | Concept | The general concept of a budget as a way to stimulate Health Behaviour. Contains both positive and negative experiences.   - Concept of FitKnip - Information of FitKnip - Overview eHealth - Reason to participate - Selection of apps by FitKnip organization - Where FitKnip found - Budget - Content apps - Content FitKnip - Participant diagnose |
|  | Prior experience eHealth | Any prior eHealth experience that motivated participants to subscribe to this project   - eHealth experience |
| Acceptability | Design | The design of the FitKnip website and the clarity of the options within FitKnip.   - Lay-out - Colors |
|  | Functionality | The functionality of the FitKnip environment. Any problems regarding access to the program, loss of function, or unexpected reactions to the program are discussed here.   - Ability to buy app |
|  | User-friendliness | The user-friendliness of the program describes the accessibility of FitKnip. It applies to factors that make the environment self-explanatory and feel understandable once confronted with the environment.   - Reminders - Usability FitKnip - Usability apps - Accessibility - Device |
|  | Privacy | The privacy of FitKnip and its applications. Describes participants’ beliefs about the safety of personal data, concerns, and points of improvement.   - Reliability - Privacy |
|  | Complexity | The effort level participants have to invest in understanding and using the FitKnip environment, combined with the level of medical information explained.   - Complexity - Log in problems - Ability to find FitKnip/app - Language |
|  | Type and number of offered eHealth applications | The themes of applications offered in FitKnip, together with the ability to find suitable applications for the participant.   - Satisfaction with Themes - Amount of apps - Matching needs - Information of apps - Categorization of apps - Overview within FitKnip - Selection of apps by participants |
| Health empowerment | Empowerment by FitKnip | Empowerment by FitKnip |
| Preliminary health outcomes | Personal barriers and facilitators | Personal barriers and facilitators in achieving health goals   - Achieving goals - Usage of FitKnip - Sharing FitKnip experiences |
|  | Effects | The effect that FitKnip has on perceived health and how different applications achieve their goals.   - Barriers/facilitators' effects - Program in app - Long term effects |
| Improvement opportunities | Improvement possibilities | Improvement possibilities |
| Future implementation | Implementation | Factors that prevent or help FitKnip from succeeding in a practical setting.   - Registration - Barriers - Facilitators - Provider of FitKnip - Costs app/FitKnip |

**Table S2.** Codebook focus group with stakeholders.

| **Theme** | **Subtheme** | **Definition and coding rules** |
| --- | --- | --- |
| Background | Background | Background of the participants   - Background |
| Concept | Concept | This theme is about the concept of FitKnip and the separate components of FitKnip   - General - Budget - Apps - Themes of apps - Target group - FitKnip environment - Quality of apps FitKnip - Selection apps - Provider FitKnip |
| Concept | Society and target population | This theme is about FitKnip and the need of the society and individuals of specific organizations and the fit with the target group and the difficulties for that target group to use FitKnip.   - Society – facilitators - Society – barriers - Participants your organization - Fit with the target group - Difficulties participants - Activation target group |
| Future implementation | Implementation by organization | This theme is about what the role of the professionals’ specific organization is in implementing FitKnip   - Role - Changes - Resources - Support |
| Future implementation | Professional specific vision and tasks | This theme is targeted at the specific vision of the professional and changes in their tasks if FitKnip were to be implemented by their organization.   - Improvement healthcare - Tasks |
| Future implementation | Implementation of FitKnip | This theme focuses on what plays a role in the successful implementation of FitKnip and who would be the organization(s) to implement FitKnip.   - Organizations involved implementation FitKnip - Organizations financing - Assessment framework - Improvements - Facilitators - Barriers - Facilitators and barriers other ehealth projects - PGO |
