# Supplementary material for: A Health App Platform Providing a Budget to Purchase Preselected Apps as an Innovative Way to Support Public Health: Qualitative Study With End Users and Other Stakeholders
Source: JMIR Form Res. 2023 Sep 29;7:e49473. doi: 10.2196/49473 (PMC10576224; doi:10.2196/49473)
Supplement: Multimedia Appendix 4 [file formative_v7i1e49473_app4.docx]

**Table S1.** Overview results acceptability.

| **Subject** | **Results** | **Quotes** |
| --- | --- | --- |
| Design | **Lay-out**  Users stated that the lay-out was fine, fresh, clear, sober, and calm.  **Colors**  The colors (white, yellow, and black) were considered neutral, sober, pretty, good, and nice. | *‘I thought the lay-out was pretty clear. That is pleasant, visually.’ Female, 66 – Focusgroup at T1*  ‘*I like the color yellow, it’s a warm enjoyable color’ Female, 27 – Focusgroup at T1* |
| User-friendliness | **Usability FitKnip**  The FitKnip platform was seen as usable, neat, and orderly.  **Reminders**  Usability could be increased if motivational reminders were added to the platform. Users want to set the frequency of the reminders themselves.  **Accessibility**  End-users indicated wanting to use one account for the platform and the apps and that the platform links to the apps directly without having to sign on again.  Some of the applications are not accessible to individuals with a visual impairment. | *‘Honestly, I thought it was an easy website’ Female, 49 – Focusgroup at T3*  *‘I bought an application. You don’t finish the program at once. Therefore it can happen that you forget about it. That is a shame. There should be a system, that if you don’t log in for a week and you didn’t finish the program, you will receive an app of mail.’ Female, 63 – Focusgroup at T3*  ‘*I think it will help if you are linked to the applications through FitKnip without having to log in again and again.*’ *Female, 49 – Focusgroup at T3* |
| Complexity | **Ability to find FitKnip/app** End-users indicated FitKnip was not easy to find since FitKnip is a website and not an application. For many end-users it was unclear where the purchased applications could be found. ‘ | *‘I think it will be beneficial to have one general screen. That it is clear which applications you purchased and which not.’*  *Female, 64 – Focusgroup at T1* |
| Type and number of offered health apps | **Satisfaction with themes**  Some end-users were satisfied with the collection of applications. A majority of end-users indicated missing applications regarding physical health and prevention, such as nutrition, sports, and exercise. End-users who had cancer or a burn-out indicated missing applications concerning mental capacity, fatigue, and consequences of cancer.    **Information of apps**  End-users indicated that they would like more information about the applications within FitKnip in order to make an informed choice. Information they missed was the time investment and the goal of the application. Information can also be offered in the form of previews, reviews or a short trial period.  **Categorization of apps** End-users indicated that next to the categorization in themes, they liked to see categorization based on target group, such as age category, level, stage of illness. Some applications fell under several themes; this led to confusion amongst end-users.  **Selection of apps by end-users**  A part of end-users suggested a personal recommendation by FitKnip based on a couple of questions/a lifestyle scan.  **Overview within FitKnip**  A part of end-users stated that the overview of FitKnip was clear; however, some other end-users thought it was not clear enough. They would like to see their budget and purchased applications at a single glance and that a distinction is made between purchased, viewed, and offered applications. | *‘I think the selection is too limited. I think it is too much targeted at mental health. A lot of applications can be added, for example on diet’ Male, 56 – Focusgroup at T1*  *‘FitKnip provided relatively limited information before purchase. Therefore it’s hard to decide if you want to buy a app of 9 euros.’*  *Male, 53 – Focusgroup at T1*  *‘For example, that next to the thematical categorization, you also have categorization based on level. Or in one category you can find all the applications for prevention. But, you need something for advanced users, especially for topics such as mindfulness and relaxation.’ Male, 56 – Focusgroup at T3*  *‘I would like a recommendation from FitKnip, based on a couple of personal questions. Like these applications suit you and we will take these out. Female, 27 – Focusgroup at T1*  *‘That you see at a single glance: this is my budget and these are the application I bought.’ Male, 58 – Focusgroup at T1* |
| Privacy | Some end-users expressed that they considered privacy as an important topic, while others did not see this as important. This varied between different applications. A part of end-users indicated seeing FitKnip as safe and trustworthy. End-users indicated experiencing less privacy in the applications offered within FitKnip. End-users indicated that transparency is needed regarding that privacy is one of the selection criteria. | *‘If I would not have believed in the privacy [of FitKnip], I would not be here. … I would not have participated in FitKnip. … I believe the provider ensures my privacy and protects my data’* |

**Table S2.** Roles in the future implementation of stakeholders viewed by end-users and stakeholders.

|  | Users |  |  | Stakeholders |  |  |
| --- | --- | --- | --- | --- | --- | --- |
| Stakeholders | Advantages providers | Disadvantages | Financing | Own role | Role other organizations | Financing |
| Health insurer | - Interest in: healthy insured person, fewer costs - Accessible, everyone has health insurance (in the Netherlands) - Reliable (for some end-users) | - Commercial interest - Reach health insurers the target group to reduce health inequality - Fear that applications will replace the care | X, since healthcare costs possibly will be reduced | - Possibility of supplementary insurance (On personal note) - Facilitate Digital platform on the website (Personal note) - Communication about FitKnip (Campaigns) |  | Financing prevention needs to be explored further |
| Employers | - Reliable - Facilitate return to work (after disease) | - Conditions - Employer no insight into data - Company-wide, not only with health problems - Preference via occupational physician instead of employer/manager |  | - |  |  |
| Primary or secondary care | - Easy to integrate into care trajectory for the patient - No commercial interest |  | X, disadvantage as this will increase healthcare costs | - FitKnip as an app on prescription, only when the platform is proven effective and approved by a GP association |  | - Other financing systems are needed, when this is going to be offered by the GP - Reimbursement via insurer |
| Patient associations | - Patient advocates - Large member base |  |  |  |  |  |
| Municipality/neighborhood | - Accessible for citizens - Aware of the needs of citizens |  | X, via current legislation and subsidies | - Community worker: possible to offer FitKnip, however with both on- and offline interventions - Municipality: Support GPs - Communication and support of other parties, such as libraries and sports clubs | - See the important role of GP, with the condition that FitKnip is effective and safe; otherwise, it can damage the reputation of the GP. | - Prevention is a joint goal, and combining budgets is a part of this. |
| App developer |  |  | X, pay to have their apps in FitKnip |  |  |  |
| Ministry of Health, Welfare and Sports (NL) |  |  |  | - Supporting role in the implementation of FitKnip - Offer FitKnip in the environment of the individual. | A combination of FitKnip providers, such as the employer, health insurer, GP, municipality, and neighborhood. | Multiple financing parties, combining budgets |
